# No correlation between acetylcholine receptor antibody concentration and individual clinical symptoms of myasthenia gravis: A systematic retrospective study involving 67 patients

**DOI:** 10.1002/brb3.2203

**Published:** 2021-06-02

**Authors:** Lulu Wang, Shumin Wang, Haonan Yang, Jiaojiao Han, Xue Zhao, Sensen Han, Yingna Zhang, Jie Lv, Jing Zhang, Mingqiang Li, Ying Ji, Shuxian Zhou, Xiaoxiao He, Hua Fang, Junhong Yang, Yunke Zhang, Qingyong Zhang, Peiyang Gao, Feng Gao

**Affiliations:** ^1^ Department of Neurology The Second Affiliated Hospital of Zhengzhou University Zhengzhou China; ^2^ Department of Neuroimmunology, Henan Institute of Medical and Pharmaceutical Sciences Zhengzhou University Zhengzhou China; ^3^ Basic Medical College Zhengzhou University Zhengzhou China; ^4^ BGI College Zhengzhou University Zhengzhou China; ^5^ Department of Encephalopathy First Affiliated Hospital of Henan University of TCM Zhengzhou China; ^6^ Myasthenia Gravis Comprehensive Diagnosis and Treatment Center Henan Provincial People’s Hospital Zhengzhou China; ^7^ Department of Clinical Medicine Xinxiang Medical University Sanquan Medical College Xinxiang China

**Keywords:** acetylcholine receptor antibodies, clinical severity, myasthenia gravis, therapy

## Abstract

**Objective:**

To investigate the correlation between acetylcholine receptor antibodies (AChR‐Ab) concentration levels and individualized clinical symptoms in patients with AChR myasthenia gravis (AChR‐MG) in China.

**Methods:**

ELISA was used to determine the concentration of AChR‐Ab in patients with MG. The Myasthenia Gravis Foundation of America (MGFA) Clinical Classification, Quantitative Myasthenia Gravis (QMG) score, and MG‐specific activities of daily living (MG‐ADL) scoring systems were used to evaluate the clinical status of patients. Spearman correlation analysis was used to determine the correlation between the AChR‐Ab concentration and clinical score. The changes in the antibody concentration and clinical score are shown in MGFA‐antibody concentration–treatment plots.

**Results:**

Autoantibody detection tests were performed in 67 patients, and their clinical scores were recorded. Forty‐nine patients received immunosuppressive therapy, 17 patients received pyridostigmine only, and 1 patient under thymectomy without any medication. The AChR‐Ab concentration correlated with the MGFA Classification in 5 (29.4%) patients in the pyridostigmine‐only group and 15 (30.6%) patients in the immunosuppressive drug group. The changes in the MGFA Classification preceded the changes in the AChR‐Ab concentration in 4 (23.5%) patients treated with pyridostigmine and 10 (20.4%) patients on immunosuppressive drugs. In patients on oral non‐steroidal immunosuppressants, the AChR‐Ab concentration changed by more than 50%, whereas the MGFA Classification did not increase. The AChR‐Ab concentration decreased in 17/32 (53.1%) patients after thymectomy, and then increased, whereas the AChR‐Ab concentration increased in 15/32 (46.9%) patients and the MGFA Classification decreased in 27/32 (81.8%) patients after thymectomy. The AChR‐Ab concentration presented a slight correlation with the corresponding MGFA, QMG, and MG‐ADL in patients with thymoma.

**Discussion:**

In the Chinese AChR‐MG population, the Changes in the AChR‐Ab concentration in individuals with AChR‐MG did not consistently correlate with the severity of clinical symptoms.

## Graphical abstract

We detected AChR‐Ab concentration at least four times per patient and performed corresponding clinical evaluations on 67 patients, and MGFA‐antibody concentration–treatment plots were drawn for each patient. We found that patients receiving oral glucocorticoid doses greater than 40 mg/d and nonsteroidal immunosuppressive drugs may have increased or decreased AChR‐Ab concentrations, and the clinical symptoms often improved to varying degrees. We did not find a consistent correlation between the changes in individual AChR‐Ab concentrations and the severity of clinical symptoms in patients.

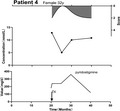

## 1 INTRODUCTION

Myasthenia gravis (MG) is an autoimmune disease in which post‐synaptic receptors at the neuromuscular junctions are inhibited by autoantibodies. It manifests as muscle weakness and fatigue (Gilhus & Verschuuren, 2015). Acetylcholine receptor antibodies (AChR‐Ab) play a pathogenic role through complementary activation, antigenic modulation, and antigenic crosslinking, leading to the loss of AChR in postsynaptic membranes (Gilhus et al., 2019; Kordas et al., 2014; Tuzun & Christadoss, 2013). AChR‐Ab can be detected in 85% of patients (Lindstrom et al., 1976), referred to as patients with AChR‐MG in the current study. Antibodies against muscle‐specific kinase (MuSK) were found in the serum of 40%–70% of patients with MG who were AChR‐Ab negative (Hoch et al., 2001; McConville et al., 2004). These patients usually present more severe symptoms, and the antibody concentration is significantly related to disease severity, in individuals and in the overall population (Bartoccioni et al., 2006). Patients without AChR‐Ab and MuSK‐Ab are called double‐negative MG (DNMG) patients. Low‐density‐lipoprotein‐receptor‐associated protein 4 (LRP4) antibodies were detected in 3 ~ 46.3% of patients with DNMG, and they are generally associated with mild clinical symptoms (Higuchi et al., 2011; Park et al., 2018; Pevzner et al., 2012). Antibodies against clustered acetylcholine receptors were detected in 38.1% of patients with MG lacking AChR‐Ab, MuSK‐Ab, and LRP4‐Ab (Cruz et al., 2015). These patients have less muscle weakness crisis and respond to treatment with cholinesterase inhibitors and immunosuppressants (Devic et al., 2014).

For patients with AChR‐MG, who comprise the largest proportion of the MG populations, it is not clear whether decreasing the serum AChR‐Ab concentration corresponds to an improved clinical score. It is considered that there is no consistent correlation between the serum AChR‐Ab concentration and clinical severity in the whole population with MG (Aurangzeb et al., 2009; Olanow et al., 1982). Some studies have suggested that the antibody concentration is relatively high in patients with severe clinical symptoms (Bilinska et al., 1999; Somnier, 1993). Regarding the changes in the AChR‐Ab concentration, studies in the US and Norway have indicated that the AChR‐Ab concentrations can help predict the clinical status of patients receiving immunosuppressive drugs in cohort studies (Heldal et al., 2014; Sanders et al., 2014). To explore the correlation between the AChR‐Ab concentration and the severity of the symptoms in individuals with AChR‐Ab in China, we compared the serum AChR‐Ab concentrations of 67 patients with AChR‐MG with their individual clinical symptoms and treatments.

## 2 MATERIALS AND METHODS

### 2.1 Patients

In this study, 67 patients with MG were screened from a pool of patients with myasthenia gravis. These patients were from the Department of Neuroimmunology, Henan Institute of Medical and Pharmaceutical Sciences, Zhengzhou University. They visited the department from April 2009 to August 2019. The inclusion criteria were as follows: (1) patients diagnosed with AChR‐MG (Gilhus & Verschuuren, 2015) and (2) serum AChR‐Ab tests performed ≥4 times. Patients who met both criteria were included in this study. The interval of repeated antibody tests was determined by the patients or physicians, and the range is 1 month to 1 year.

To eliminate the interference of MuSK‐Ab and LRP4‐Ab on the analysis, the detection of MG autoantibodies included the detection of AChR‐Ab, MuSK‐Ab, and LRP4‐Ab. Twenty‐four hours after the patient discontinued the administration of pyridostigmine, blood samples were collected and centrifuged, and the upper serum was collected, stored at 4℃, and tested for AChR‐Ab within 1 week. The remaining serum was stored at −80℃ indefinitely after being packaged for future detection of MuSK‐Ab and LRP4‐Ab. To reduce experimental error, serum samples were thawed ≦ 2 times.

All clinical investigations were conducted in accordance with the principles in the Helsinki Declaration, and the study was approved by the Medical Ethics Committee of Henan Institute of Medical and Pharmaceutical Sciences, Zhengzhou University, and the committee waived the need for informed consent of patients.

### 2.2 Autoantibody testing

#### 2.2.1 ELISA for binding AChR‐Ab

The AChR‐Ab ELISA kits (RSR Ltd., Cardiff, UK) were used to measure binding AChR‐Ab concentration according to the manufacturer's instructions. Firstly, the inhibition rate of the test sample was calculated according using the following formula: inhibition rate =100×[1‐ absorbance of the test sample at 450 nm/negative control (N) absorbance at 450 nm]. Secondly, the AChR‐Ab concentration was calculated based on the inhibition rate: AChR‐Ab concentration =0.2 × 2 ^(0^^.^^067×% inhibition rate of the test sample)^.

#### 2.2.2 Cell‐based assays for MuSK‐Ab and LRP4‐Ab

Cell‐based assays were used to detect MuSK‐Ab and LRP4‐Ab. These test methods have been described in our previous report (Li et al., 2019).

### 2.3 Clinical evaluation

The clinical evaluation of patients was performed on the same day their serum sample was obtained. Evaluation methods included the Myasthenia Gravis Foundation Of America (MGFA) clinical classification, Quantitative Myasthenia Gravis (QMG) score, and MG‐specific activities of daily living (MG‐ADL) scoring systems. The MGFA clinical classification and MG‐ADL scoring systems were used to evaluated 30 patients younger than 18 years. The MGFA classification was used to assess the clinical status of patients (Jaretzki et al., 2000). Taking into account the objective law of changes in clinical symptoms, the MGFA classification is divided into five classes and in our study, no distinction was made between subclasses a or b. The MGFA scores were recorded as 0 (0 for asymptomatic patients), 1 (Class I), 2 (Class IIa, IIb), 3 (Class IIIa, IIIb), 4 (Class IVa, IVb), and 5 (Class V). The QMG score has been used in prospective studies on MG therapy to assess the clinical changes in patients (Bedlack et al., 2005). MG‐ADL is closely related to QMG score, expresses the subjective feelings of patients, and has been used as an indicator of secondary efficacy in clinical trials (Wolfe et al., 1999).

### 2.4 Statistics

Statistical analyses were performed using IBM Statistical Package for the Social Sciences (SPSS) version 21, and GraphPad Prism 8 was used to create all figures. The correlation analyses comparing the AChR‐Ab concentration with age and the three clinical status scores (MGFA, QMG, and MG‐ADL) were performed using Spearman's test. Mann–Whitney U test was used to analyze the differences in the AChR‐Ab concentration between the sexes. The differences in antibody changes before and after surgery were analyzed using Wilcoxon signed‐rank test and Friedman test. In addition, we generated MGFA‐antibody concentration–treatment plot for each patient.

## 3 RESULTS

### 3.1 Demographics

The AChR‐Ab, MuSK‐Ab, and LRP4‐Ab concentrations were assessed in 67 patients with MG. In total, 399 autoantibody tests were performed, in all of which MuSK‐Ab and LRP4‐Ab were negative. At the same time, these patients were clinically evaluated based on the MGFA, QMG, and MG‐ADL scores. Of the 67 patients with MG, 39 were female and 28 male. The age of disease onset was 2–70 years old (mean =26 years old), and the follow‐up time was 0.5–10 years (mean =3.4 years) (Table 1). Patients were tested for AChR‐Ab concentration 4–14 times. Patients with the highest number of serum antibody tests (14 times) were followed up for 5.25 years.

**TABLE 1:** Basic information of all patients (*n* = 67)

	Total	EOMG	LOMG
Sex			
Male	28	20	8
Female	39	34	5
M:F^a^	1:1.39	1:1.70	1:0.63
Presenting symptom			
Ocular	60 (89.6%)	49 (90.7%)	11 (84.6%)
Bulbar	3 (4.5%)	2 (3.6%)	1 (7.7%)
Limb	1 (1.5%)	1 (1.9%)	0 (0.0%)
Cervical	2 (3.0%)	1 (1.9%)	1 (7.7%)
Respiratory	1 (1.5%)	1 (1.9%)	0 (0.0%)
Neostigmine test			
Total	50	42	8
Positive	45 (90%)	37 (88.1%)	8 (100%)
RNS test			
Total	31	24	7
Positive	20 (64.5%)	16 (66.7%)	4 (57.1%)
Thymic status^b^			
Total	56	45	11
Involuted	27 (48.2%)	22 (48.9%)	5 (45.5%)
Hyperplasia	15 (26.8%)	15 (33.3%)	0 (0.0%)
Thymoma	14 (25.0%)	8 (17.8%)	6 (54.5%)
Treatment			
pyridostigmine‐only	17	12	5
Immunosuppressive	49	41	8
prednisone	47	39	8
cyclosporine A	2	2	0
thymectomy	35	26	9

Note

EOMG, early‐onset myasthenia gravis (MG patients with first onset age <50 years); LOMG, Late‐onset myasthenia gravis (MG patients with first onset age ≥50 years); RNS, repetitive nerve stimulation.

^a^ Ratio of males to females.

^b^ Thymus status certified by chest CT.

**FIGURE 1:**
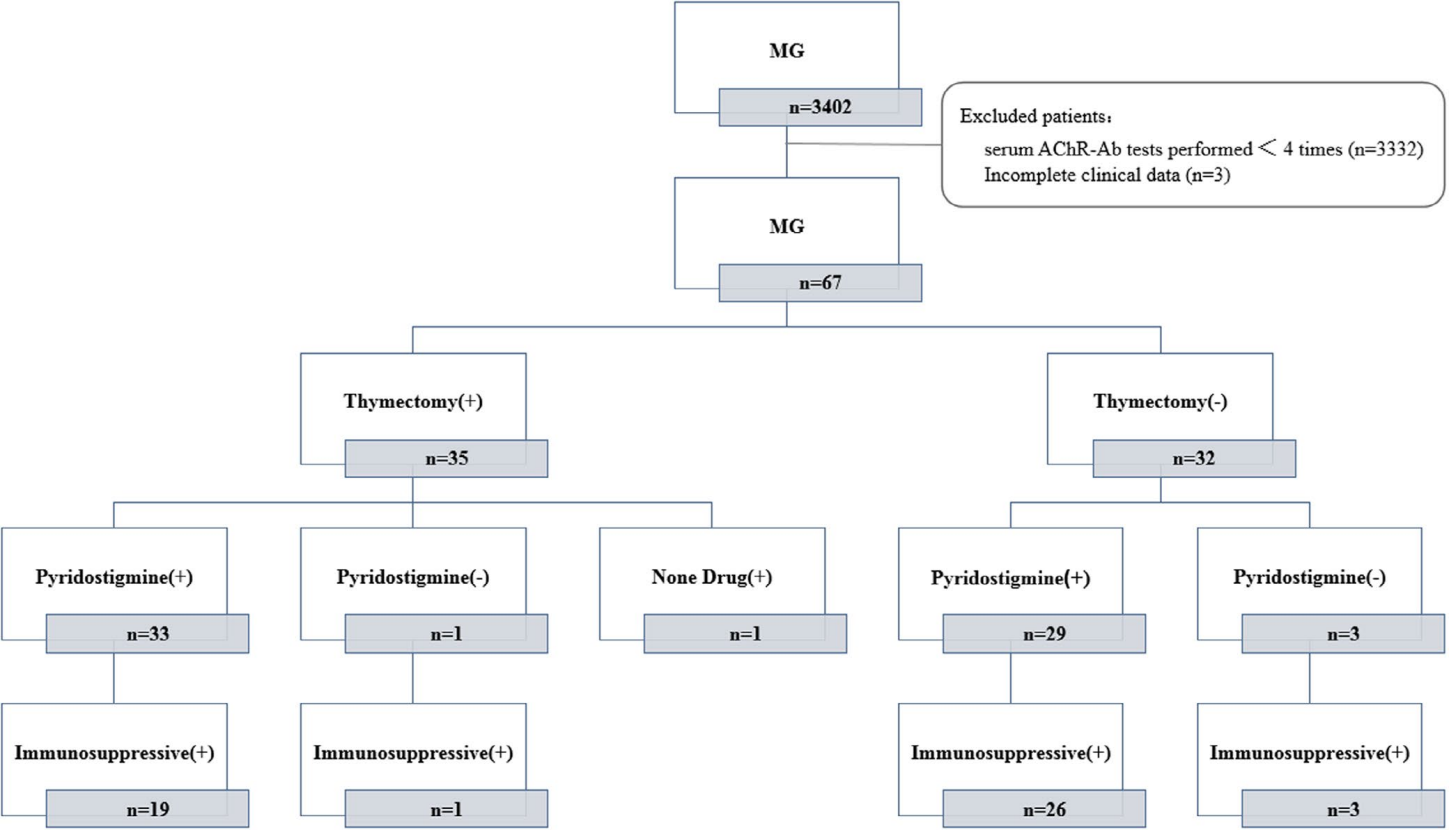
Specific treatment measures of patients

Of the 67 patients with MG, ocular muscle weakness was the first symptom in 60 patients, and of the remaining 7 patients, 3 presented with bulbar muscle weakness, 1 with limb band weakness, 2 with neck muscle weakness, and 1 with respiratory muscle weakness. Forty‐nine patients received immunosuppressive therapy: 47 received prednisone with or without pyridostigmine orally, 1 patient received cyclosporine A combined with pyridostigmine, and 1 patient received only cyclosporine A. Seventeen patients received a single treatment of pyridostigmine. One patient underwent thymectomy without any medication. Of these 67 patients, 35 had undergone thymectomy, accounting for 52.2% of the patient cohort (Figure 1). In the last follow‐up of each patient, the post‐intervention status (PIS) was as follows: 7 cases of complete stable remission (CSR), 11 cases of pharmacological remission (PR), 27 cases of minimal manifestations (MM), 16 Improved cases, 1 Unchanged case, 1 Worse case, and 4 Exacerbation cases, and 67% of the patients achieved MM status or better.

### 3.2 AChR‐Ab concentration levels and clinical status relevance

There were no significant correlations between the AChR‐Ab concentration and the corresponding MGFA, QMG, and MG‐ADL scores of patients (*r*_s_ = .29, *p* < .05; *r*_s_ = .17, *p* = .01; *r*_s_ = .23, *p* < .05). In patients with thymectomy, irrespective of whether they received pyridostigmine only or immunosuppressive drugs, there was no correlation between the AChR‐Ab concentration and MGFA, QMG, and MG‐ADL scores. In patients with thymoma, an analysis of data 13 times for AChR‐Ab detection in 11 patients before thymectomy showed that the AChR‐Ab concentration and MGFA score have a moderate correlation (*r*_s_ = .67, *p* < .05). There was no correlation between the AChR‐Ab concentration and QMG and MG‐ADL (*r*_s_ = .55, *p* = .05; *r*_s_ = .48, *p* = .10). An analysis of the data 72 times in 14 patients after thymectomy showed that the AChR‐Ab concentration slightly correlated with the MGFA, QMG, and MG‐ADL scores (*r*_s_ = .47, *p* < .05; *r*_s_ = .46, *p* < .05; *r*_s_ = .38, *p* .05). There was no significant difference in the AChR‐Ab concentration between sexes (*p* = .86), and there was only a slight correlation with age (*r*_s_ = .39, *p* < .05).

### 3.3 AChR‐Ab concentration levels and consistency with individual clinical symptoms

#### 3.3.1 Correlation between the AChR‐Ab concentration and individualized clinical scores

**FIGURE 2:**
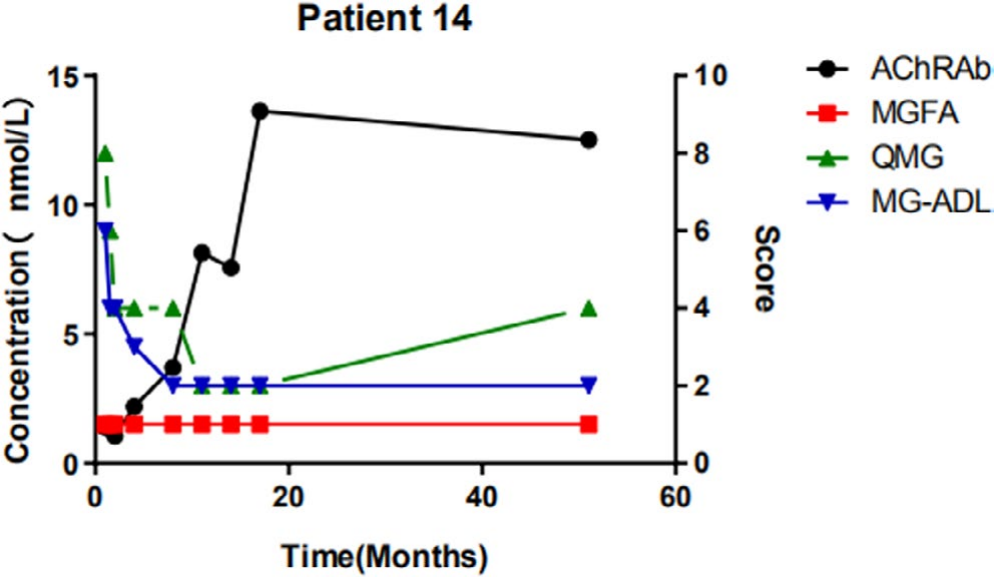
Changes in AChR‐Ab concentration and clinical symptoms of patients with MG over time. Female, 24 years old, QMG scores and AChR‐Ab concentrations were largely matched, but her MGFA and MG‐ADL scores did not change with AChR‐Ab concentration

For 12 patients who had more than seven repeated AChR‐Ab detection tests, Spearman's test was used to analyzed the correlation between the AChR‐Ab concentration and clinical score. Only in patient 14, the AChR‐Ab concentrations correlated with the clinical scores. In this patient, when the QMG and MG‐ADL levels decreased, the antibody concentration increased (Figure 2).

#### 3.3.2 AChR‐Ab concentration changes during the course of disease

**FIGURE 3:**
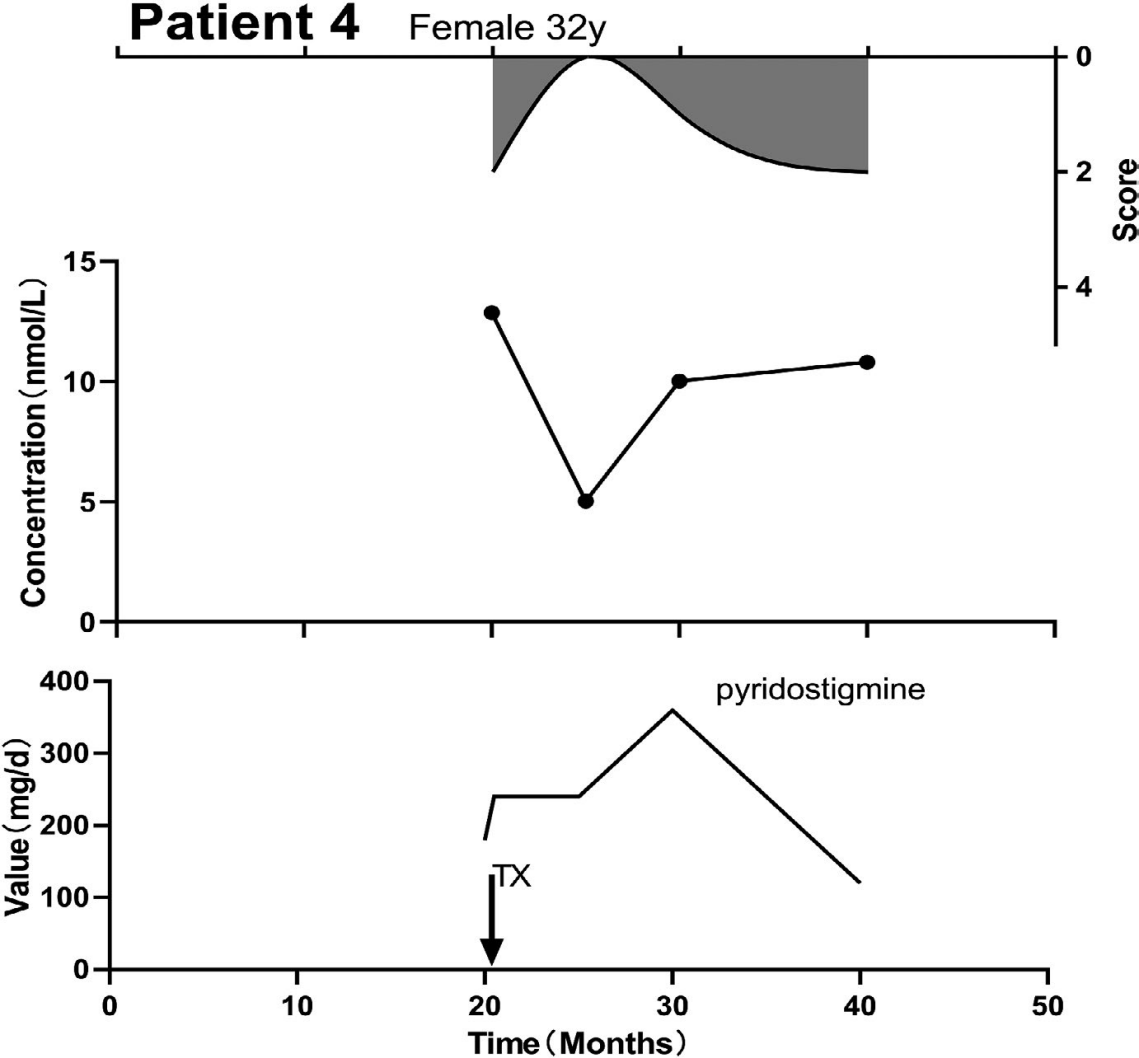
Correlation between AChR‐Ab levels and MGFA score over time in a representative patient. The shaded surface represents the MGFA score. The middle graph displays AChR‐Ab concentration (nmol/L). The lower line chart represents the treatment. The x‐axis reports the patient's MG course from the time of onset, while y‐axis reports MGFA score, AChR‐Ab concentration, and drug dose and these applies to all the figures. TX: Thymectomy. Female, 32 years old. Two years after onset, she sought medical treatment due to ptosis and an inability to chew. Testing the patient for AChR‐Ab concentrations began at this point, 20 months after disease onset. Her MGFA was IIb and her AChR‐Ab concentrations were at their highest level in the course of MG progression. After administration of oral pyridostigmine (180 mg/d) improved her symptoms, a thymectomy was performed immediately. After an oral pyridostigmine course of 240 mg/d, the symptoms disappeared, and the AChR‐Ab concentration decreased. More than 9 months after thymectomy, the patient reported the reappearance of ptosis, and her MGFA score (I) and AChR‐Ab concentrations had increased. The prescribed dosage of pyridostigmine was adjusted to 360 mg/d without any reduction in symptoms. At the last review, the patient's vision was blurred, her right hand was weak after activity, and her MGFA (IIa) and AChR‐Ab concentrations had further increased

Of the 67 patients, the AChR‐Ab concentration in 28 (41.8%) patients was not consistent with their corresponding MGFA classification. Twenty (29.8%) patients exhibited a correlation between the AChR‐Ab concentration and MGFA score over time (Figure 3). We believe that the AChR‐Ab concentration is related to MGFA score in the following two scenarios: when the maximum and minimum score of MGFA during the stable period are less than 50% or antibody tests were negative; and the shape of change in MGFA was consistent with that in antibody concentration. In 15 (22.4%) cases, the changes in the MGFA score were observed before changes in the AChR‐Ab concentration, and 4 (6.0%) cases showed changes in the AChR‐Ab concentration before a change in the MGFA score.

### 3.4 Effects of pyridostigmine‐only treatment on AChR‐Ab concentration

**FIGURE 4:**
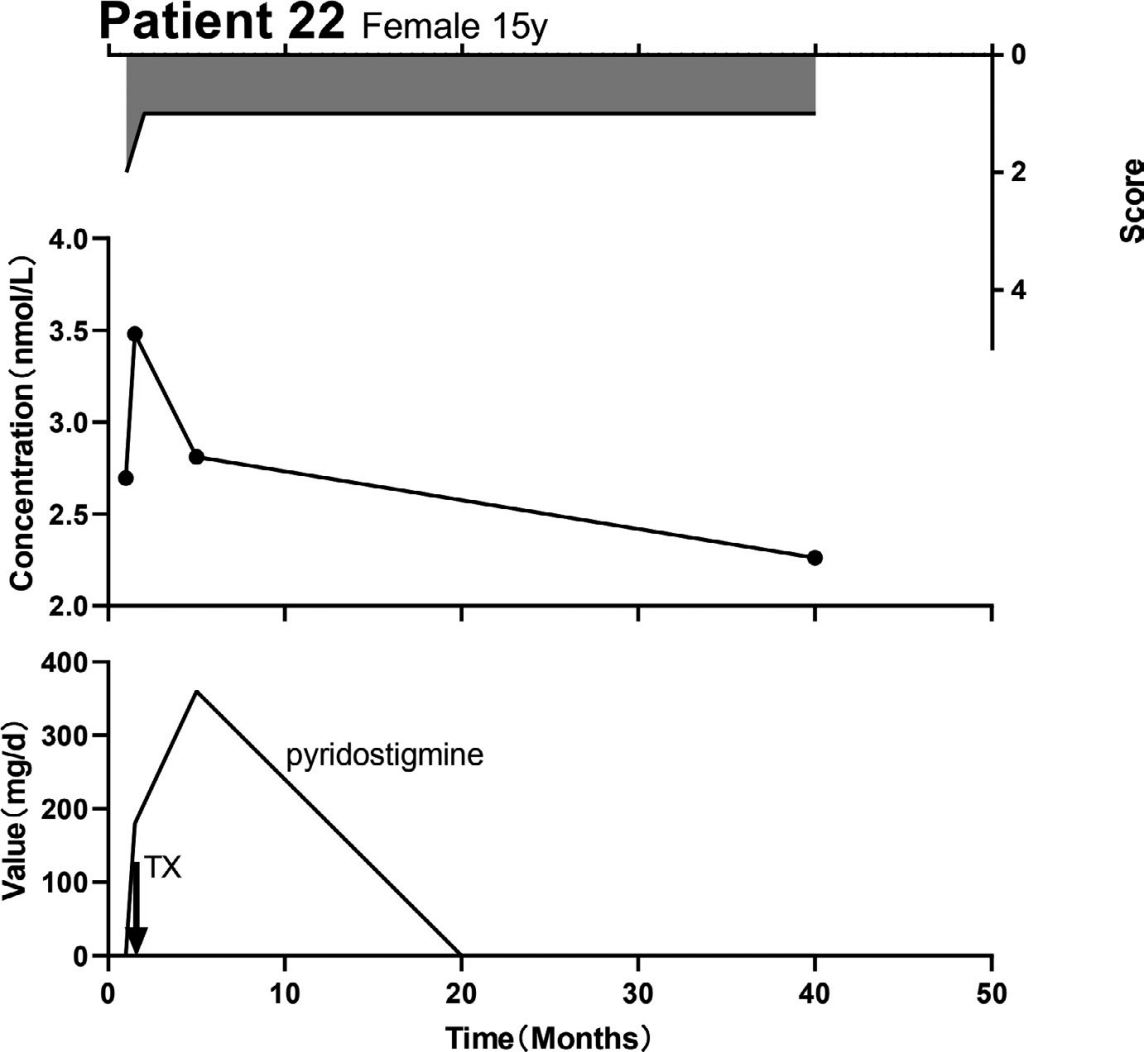
Changes in individual MGFA scores prior to changes in AChR‐Ab concentration. Female, 15 years old. The patient presented with ptosis and weakness in chewing. The MGFA score (IIb) and AChR‐Ab concentration were not particularly high during her follow‐up. A dose of oral pyridostigmine (180 mg/d) improved her symptoms and a thymectomy was performed immediately. Postoperatively, the patient's symptoms improved, and her MGFA score (I) and AChR‐Ab concentration increased. After 2 weeks, her AChR‐Ab concentration increased. When the AChR‐Ab concentration of the patient was tested for a third time, there was only a slight drooping eyelid

Seventeen patients received treatment with pyridostigmine only. In this group, 8 (47.1%) did not show a correlation between their AChR‐Ab concentration and MGFA score, while whereas in 5 patients (29.4%), the AChR‐Ab concentration positively correlated with their MGFA scores. Figure 3 presents a representative case of this phenomenon. Four cases (23.5%) showed changes in the MGFA score before changes in the AChR‐Ab concentration (Figure 4). Of the 17 patients on pyridostigmine‐only treatment, the serum antibody tests for more than 2 years were negative in two patients (11.8%).

### 3.5 Effects of immunosuppressive drugs on AChR‐Ab concentration

**FIGURE 5:**
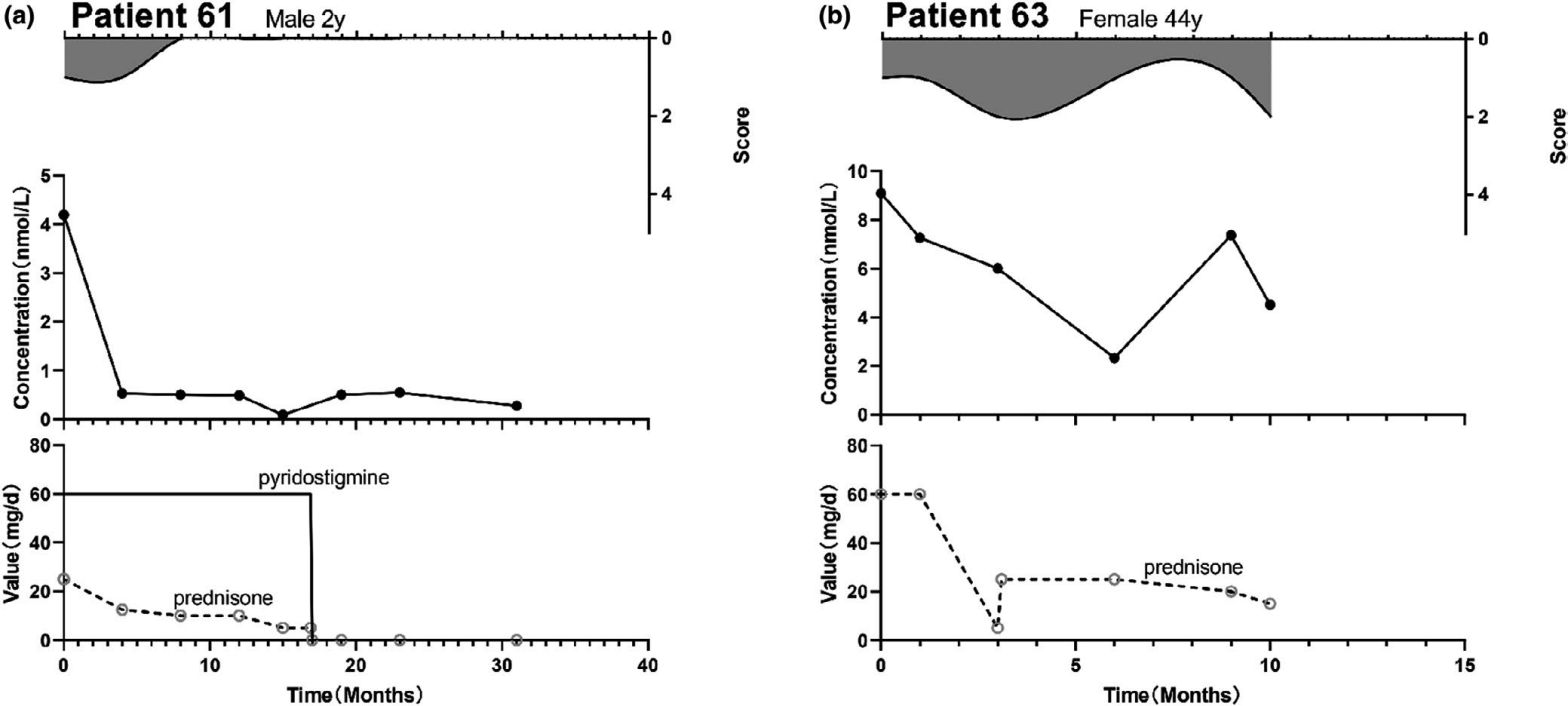
MGFA‐antibody concentration–treatment plots for patients who received immunosuppressive therapy. (a) Male, 2 years old. The patient presented with ptosis, and both the MGFA (I) score and AChR‐Ab concentration were at their highest values in the course of the disease. With the administration of oral pyridostigmine (60 mg/d) and prednisone (25 mg/d), the symptoms gradually disappeared and AChR‐Ab concentration showed a substantial initial decrease, followed by maintenance of a relatively stable low concentration. (b) Female, 44 years old. The patient presented with ptosis and diplopia. She had an MGFA of Ⅰ and her AChR‐Ab concentration was at its highest point in the course of the disease. After starting on, an oral prednisone dose of 60 mg/d, her MGFA score remained unchanged (although her MG‐ADL score decreased from 6 to 2), and AChR‐Ab concentration decreased significantly (21.1%). When prednisone was reduced to 5 mg/d, the patient developed a cough when drinking, her MGFA increased to IIb, but her AChR‐Ab concentration continued to decrease. When we adjusted the prednisone dose to 25 mg/d, the symptoms reduced. The MGFA (Ⅰ) score decreased and AChR‐Ab concentration continued to decrease

Forty‐nine patients received immunosuppressive therapy. In 20 patients (40.8%), there was no correlation between the AChR‐Ab concentration and MGFA score. In 15 patients (30.6%), there was a consistent correlation between the AChR‐Ab concentration and MGFA score. In 10 patients (20.4%), the change in the MGFA score preceded the change in the AChR‐Ab concentration. Furthermore, the change in the AChR‐Ab concentration preceded the change in the MGFA score in 4 cases (8.2%). In nine patients (14.3%), we observed that the AChR‐Ab test result was negative for 0.25–3 years (Figure 5a).

#### 3.5.1 Effect of prednisone on the change in AChR‐Ab concentration

Forty‐seven patients received oral prednisone medication. For 34 patients, the dosage of prednisone was less than 40 mg/d, and 13 patients were administered prednisone at a dose of 40 mg/d or more. In the latter group, Spearman analysis showed no correlation between the AChR‐Ab concentration and clinical score. Meanwhile, in six patients (46.2%), the AChR‐Ab concentration decreased by more than 15% (Figure 5b), and the remaining 8 patients did not show a significant decrease in the AChR‐Ab concentration in disease development graphs. Among the 33 patients on prednisone at a dose less than 40 mg/d, we did not find a significant change in the AChR‐Ab concentration.

**FIGURE 6:**
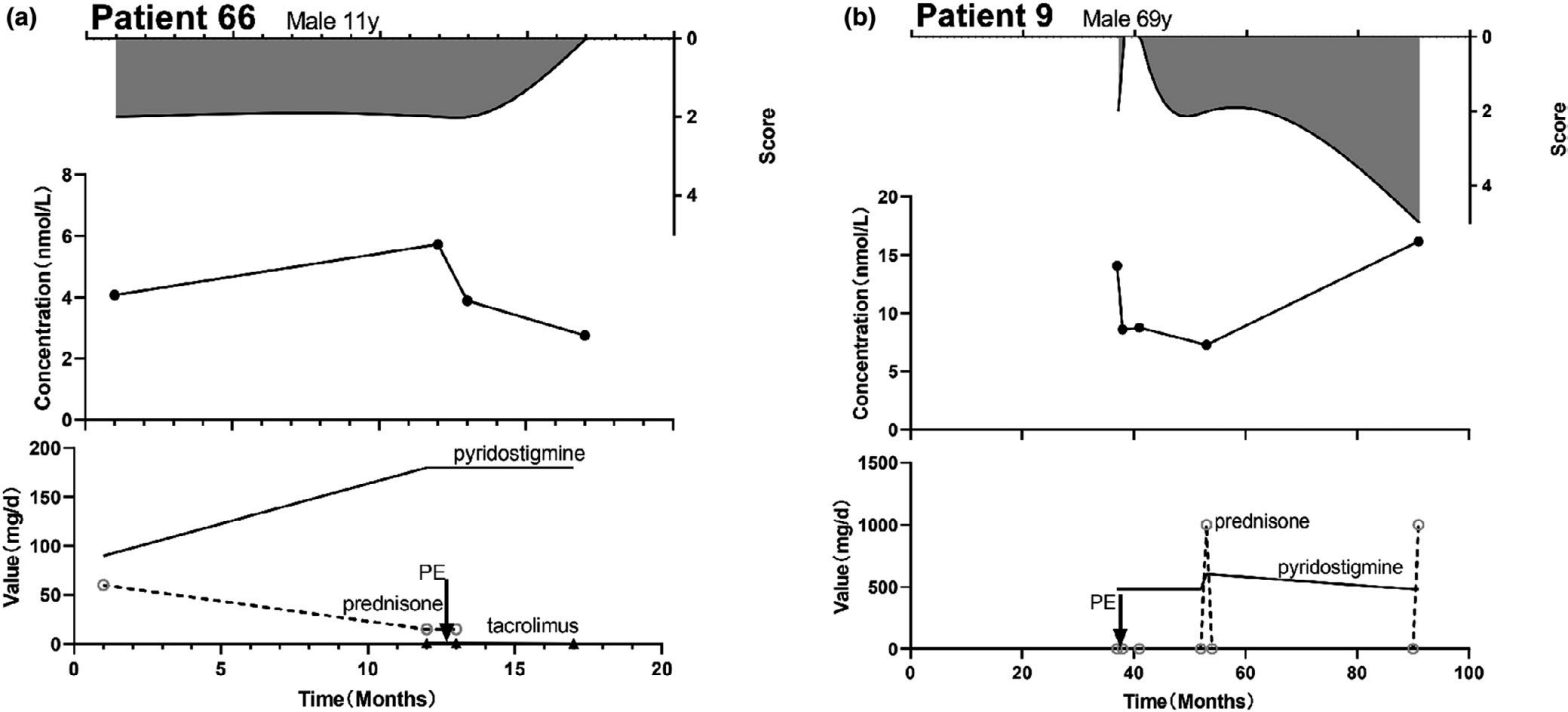
MGFA‐antibody concentration–treatment plots for patients with PE. PE: Plasma exchange. (a) The patient developed ptosis and had difficulty swallowing. He was referred to us with MGFA Ⅱa. Administration of oral pyridostigmine (90 mg/d) and prednisone (60 mg/d) had no effect on his MGFA score, although his MG‐ADL score decreased from 6 to 4 and his AChR‐Ab concentration increased. However, when the treatment regimen was adjusted to a dose of 180 mg/d pyridostigmine, 15 mg/d prednisone, and 0.9 mg/d tacrolimus, the patient's AChR‐Ab concentration decreased by 51.9% and his clinical symptoms disappeared. (b) Three years after MG onset, the patient sought medical attention for ptosis and dysphagia. His clinical scores were: MGFA Ⅱb, QMG 10, and MG‐ADL 3. He received an oral course of pyridostigmine and 8 plasma exchanges. His AChR‐Ab concentration decreased by 38.9% and his symptoms disappeared. One year later, he had difficulty swallowing due to a cold, accompanied by dyspnea. He received pyridostigmine (600 mg/d) and a shock treatment of prednisone 1,000 mg/d, after which his symptoms were reduced, along with his clinical scores. At the last recorded examination, the patient had a muscle weakness crisis

Of the nine patients who used non‐steroidal immunosuppressive drugs, three were treated with these drugs for less than 4 months. As it takes several months for patients tconcentration and MGFA score ino respond to non‐steroidal immunosuppressive drugs (Gilhus et al., 2019), we did not analyze patients who used them for less than 6 months. The correlation analysis of the remaining six patients showed no correlation between the antibody concentration and the MGFA, QMG, and MG‐ADL scores. Furthermore, we found that six patients experienced an increases or a decreases in the AChR‐Ab concentration while taking oral immunosuppressant drugs, with a rate of change greater than 50% and no increase in the MGFA score. There was no significant correlation between the AChR‐Ab concentration and MGFA score in five patients. Only 1 patient exhibited a correlation between antibody concentration and MGFA score, and this patient received plasma exchange (PE) therapy concurrently (Figure 6a).

### 3.6 Effects of plasma exchange on AChR‐Ab antibody concentration

Patient 66, a male subject, exhibited ptosis when he was 11 years old. He developed respiratory muscle weakness 1 year after the onset of disease and received plasma exchange therapy. After 2 consecutive plasma exchanges over 4 days, his AChR‐Ab concentration decreased from 5.726 nmol/L to 3.884 nmol/L. There was no change in his MGFA classification (maintained at Ⅱa), but his MG‐ADL score decreased from 4 to 2, following which, the patient's symptoms gradually disappeared (Figure 6a).

Patient 9 first had his AChR‐Ab concentration assessed 3 years after the onset of symptoms. At that time, the symptoms mainly manifested as bulbar muscle weakness. He received 8 plasma exchanges in 1 month. After plasma exchange therapy, his AChR‐Ab concentration decreased from 14.082 nmol/L to 8.599 nmol/L, and his clinical symptoms completely disappeared. His MG‐ADL score also decreased from 3 to 0, as shown in Figure 6b.

### 3.7 Effects of thymectomy on the AChR‐Ab concentration

#### 3.7.1 Variation in the AChR‐Ab concentration after thymectomy

**FIGURE 7:**
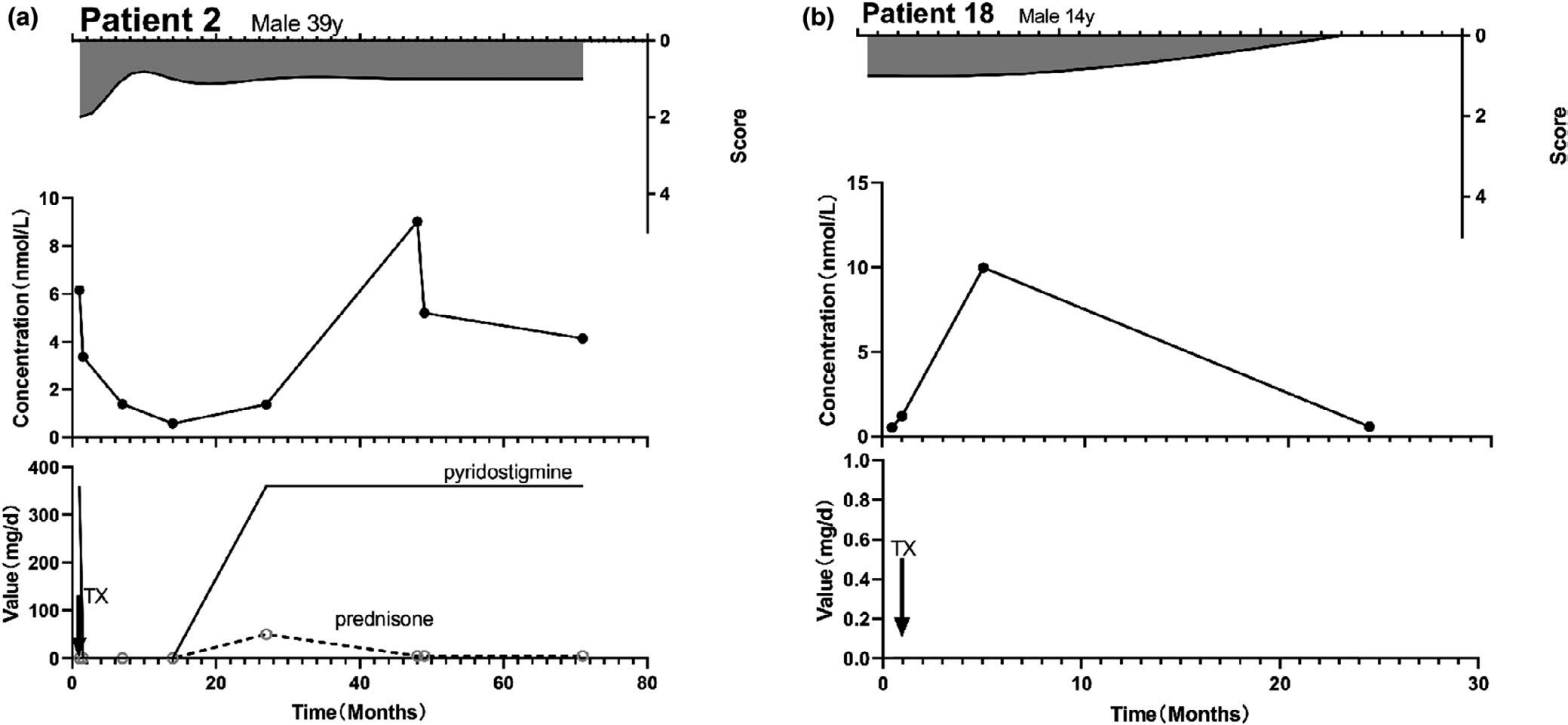
MGFA‐antibody concentration–treatment plots for patients with thymectomy. (a) In a middle‐aged male patient, AChR‐Ab concentration decreased after thymectomy, and the symptoms were reduced. After 1 year, his AChR‐Ab concentration showed an increase, and his symptoms did not change significantly. (b) In an adolescent male patient, the AChR‐Ab concentration continued to increase after surgery. Over time, his symptoms gradually disappeared, AChR‐Ab concentration decreased after 4 months, and there was no MG manifestation (this patient did not take any drugs for the management of MG)

Thirty‐five patients underwent thymectomy, and 32 procedures were performed during the follow‐up period. After thymectomy within a limited time, the AChR‐Ab concentration decreased in 17 (53.1) patients and increased in 15 patients (46.9%). In the 17 patients who exhibited a decrease in the AChR‐Ab concentration, the concentration decreased for 1 month in 9 patients (52.9%), and for 2–36 months in the remaining 8 patients. Thereafter, we observed that the AChR‐Ab concentration in 12 patients increased and exceeded their preoperative antibody level (Figure 7a). In the 17 patients who exhibited a decrease in the AChR‐Ab concentration, the MGFA scores decreased in 14 patients (82.4%) and not change in 3 patients (17.6%). In the 15 patients with increased AChR‐Ab concentration, 9 patients (60.0%) showed an increased for 1 month, and 6 patients for 2–66 months (Figure 7b). Meanwhile, the MGFA score decreased in 13 patients (86.7%) and did not change in 2 patients (13.3%). Regarding oral medications, 14 of the 35 patients took oral pyridostigmine only, 20 patients received immunosuppressive therapy, and 1 patient did not take any medication. The AChR‐Ab concentration in 4/14 (0.29%) and 5/20 (0.25%) patients in the pyridostigmine and immunosuppressive drug groups was consistent with the MGFA score; there was no significant difference between the groups. Of the 35 patients who undergoing thymectomy, 13 patients had thymus pathological data, 8 (61.5%) patients had thymoma, and 38.5% of patients did not have thymoma.

#### 3.7.2 Difference analysis of the AChR‐Ab concentration before and after thymectomy

Of the 35 patients who underwent thymectomy, we could collect relevant data of 32 patients before and after surgery. Statistical analysis of the AChR‐Ab concentrations before and after thymectomy was performed using these data and a Wilcoxon signed‐rank test showed that there were no significant differences. Of these patients, 15 had the AChR‐Ab concentration data that could be traced back to 1 week post‐surgery. There was no significant differences between AChR‐Ab concentration before and 1 week after the surgery.

## 4 DISCUSSION

This retrospective study, we analyzed the correlation between the serum AChR‐Ab concentration and clinical status in 67 patients with AChR‐MG. The patients were tested more than 4 times for antibody concentrations, providing more reliable results for data analysis. We generated MGFA‐antibody concentration–treatment plots to visualize the changes in the AChR‐Ab concentration and clinical scores during the course of the disease. In this cohort, the proportion of women was higher in the early‐onset myasthenia gravis (EOMG) group, whereas the proportion of men was higher in the late‐onset myasthenia gravis (LOMG) cohort, consistent with the demographic characteristics of MG in China (Fan et al., 2019). A study originating in the United Kingdom suggested that compared with EOMG, thymoma comprises a higher proportion of the LOMG population than EOMG (Bokoliya & Patil, 2019), and this feature was also observed in our study population.

In patients with AChR‐Ab positive MG, there is no consensus on whether there is a correlation between the AChR‐Ab concentration and MG severity. It has been suggested that the AChR‐Ab concentration is related to the clinically diagnosed form of muscle weakness, that is, the more severe the symptoms of muscle weakness, the higher the antibody concentration (Bilinska et al., 1999). However, other studies have indicated that the AChR‐Ab concentration in patients with MG is not related to the severity of the disease (Drachman et al., 1982). No correlation was found between the concentration of AChR‐Ab in patients with MG and the severity of clinical symptoms in our study cohort. However, the AChR main immunogenic region (MIR) antibody analysis reveals that the MIR‐Ab concentration has a good correlation with MG severity (Masuda et al., 2012). An analysis of factors influencing elevated AChR‐Ab concentration in patients showed that patients with LOMG are more likely to have elevated antibody concentrations (Iwasa et al., 2014). Our overall study of patients revealed a slight correlation between the antibody concentration and age of the patient.

When studying the individual disease course in patients treated with pyridostigmine alone, continuous measurement of serum AChR‐Ab concentration has been an effective method to track the changes in patients with MG (Oosterhuis et al., 1983). In our study cohort, there was no correlation between AChR‐Ab concentration and disease progression in patients with MG, and might be related to the fact that the MGFA scores of patients we included were mostly Ⅱa/Ⅱb. A correlation has been proposed between serum AChR‐Ab concentration and MGFA classification in patients with MG who have received immunosuppressive drugs (Heldal et al., 2014). A repeated assessment of AChR‐Ab concentration can help predict the clinical status of patients undergoing immunosuppressive therapy. However, there was no correlation between the AChR‐Ab concentration and disease severity in the overall group of patients administered immunosuppressive drugs in our study. In patients administered prednisone at doses higher than 40 mg/d, the serum AChR‐Ab concentration significantly decreased and the disease severity did not increase or decrease uniformly. Higher doses of prednisone and longer duration of treatment do not ensure a better prognosis, and the early administration of a combination of low‐dose prednisone with other treatment options can help achieve treatment goals sooner (Imai et al., 2015, 2018). Therefore, we do not recommend the use of glucocorticoids at doses higher than 40 mg/d for extended periods. Palace, J found that patients on non‐steroidal immunosuppressive drugs showed a persistent decrease in the serum AChR‐Ab concentration in an RCT study (Palace et al., 1998). In this study, we found that there is no correlation between the changes in antibody concentration and clinical symptoms in patients receiving non‐steroidal immunosuppressive drugs. This might be related to the small number of observations. However, the MGFA‐antibody concentration–treatment plots showed that the AChR‐Ab concentration of these patients fluctuated, but their MGFA classification exhibited a downward trend.

Rajesh, K and Carandina‐Maffeis, R have have shown that plasma exchange might improve short‐term prognosis in patients with MG (Carandina‐Maffeis et al., 2004; Kumar et al., 2015), wherese, Newsom‐Davis, J reported that the AChR‐Ab concentration negatively correlates with muscle strength after plasma exchange (Newsom‐Davis et al., 1978). Usmani, A found that LOMG and male patients are more likely to have symptomatic relief after using plasma exchange, and antibody titers may be an effective way to monitor the plasma exchange response of patients with MG (Usmani et al., 2019). The two patients who received plasma exchange treatment in our study showed reduced AChR‐Ab concentration and improved clinical symptoms after treatment. Interestingly, one patients was an elderly man, and his clinical symptoms were temporarily relieved after receiving plasma exchange therapy.

In our cohort, 84.4% of patients benefited from thymectomy, whereas in a recent study on pre‐ and post‐thymectomy MG, only 41.0% of patients benefited from surgery (Bokoliya & Patil, 2019). There was no clear trend regarding the change in the AChR‐Ab concentration after thymectomy. Some studies have suggested that the AChR‐Ab concentration decreases in patients with MG without thymoma, but there were no predictions regarding MG patients with thymoma (Kim et al., 2018). We found that thymomas are more common in patients with increased antibody concentration after thymectomy.

Studies have reported that 0.97% to 9.1% of patients with thymoma have symptoms of MG after thymectomy. Although these patients report symptoms of MG, they are non‐specific (Kondo & Monden, 2005; Nakajima et al., 2008; Sun et al., 2011). One of the patients included in the present study had symptoms of MG after undergoing thymectomy for thymoma. The clinical symptoms of the patient improved after receiving medication.

One of the limitations of our study is that it was a retrospective study. Moreover, most of the patients in our study cohort had MGFA classifications of IIa/IIb. The small number of patients with severe symptoms might have affect the analysis results of antibody concentration in disease progression in patients with severe illness. Only a few patients used non‐steroidal immunosuppressive drugs and the results would be more reliable if more patients from this population were included. Additionally, several patients began multiple treatments concurrently, which might have affect our analysis of the effect of a single treatment on the changes in antibody concentration and clinical status. Due to the lack of specific surgical data for patients undergoing thymectomy, we were unable to analyze whether different pathology types had an effect on the changes in the AChR‐Ab concentration.

## 5 CONCLUSION

In our study, we observed that AChR‐Ab concentration might be related to age in Chinese patients. Patients receiving oral glucocorticoid at doses higher than 40 mg/d may have increased or decreased AChR‐Ab concentrations, and their clinical symptoms often improve to varying degrees. In patients receiving non‐steroidal immunosuppressive drugs, the AChR‐Ab concentration presented a slight correlation with clinical scores. We did not find a consistent correlation between the changes in individual AChR‐Ab concentrations and the severity of clinical symptoms in patients with MG. Further research on AChR α subunit MIR antibodies (Masuda et al., 2012), other subunit autoantibodies (Koneczny & Herbst, 2019), and even new biomarkers such as exosomes may help monitor the severity of clinical symptoms.

## CONFLICTS OF INTEREST

None of the authors have any conflict of interest to disclose.

## ETHICAL APPROVAL

We confirm that we have read the Journal's position on issues involved in ethical publication and affirm that this report is consistent with those guidelines.

### PEER REVIEW

The peer review history for this article is available at https://publons.com/publon/10.1002/brb3.2203.
